# Supplementary figures and images for: Pediatric Exposures Associated with Caffeine Energy Products Reported to United States Poison Centers, 2011–2023
Source: J Med Toxicol. 2025 Jan 31;21(2):241–52. doi: 10.1007/s13181-025-01057-w (PMC11933505; doi:10.1007/s13181-025-01057-w)

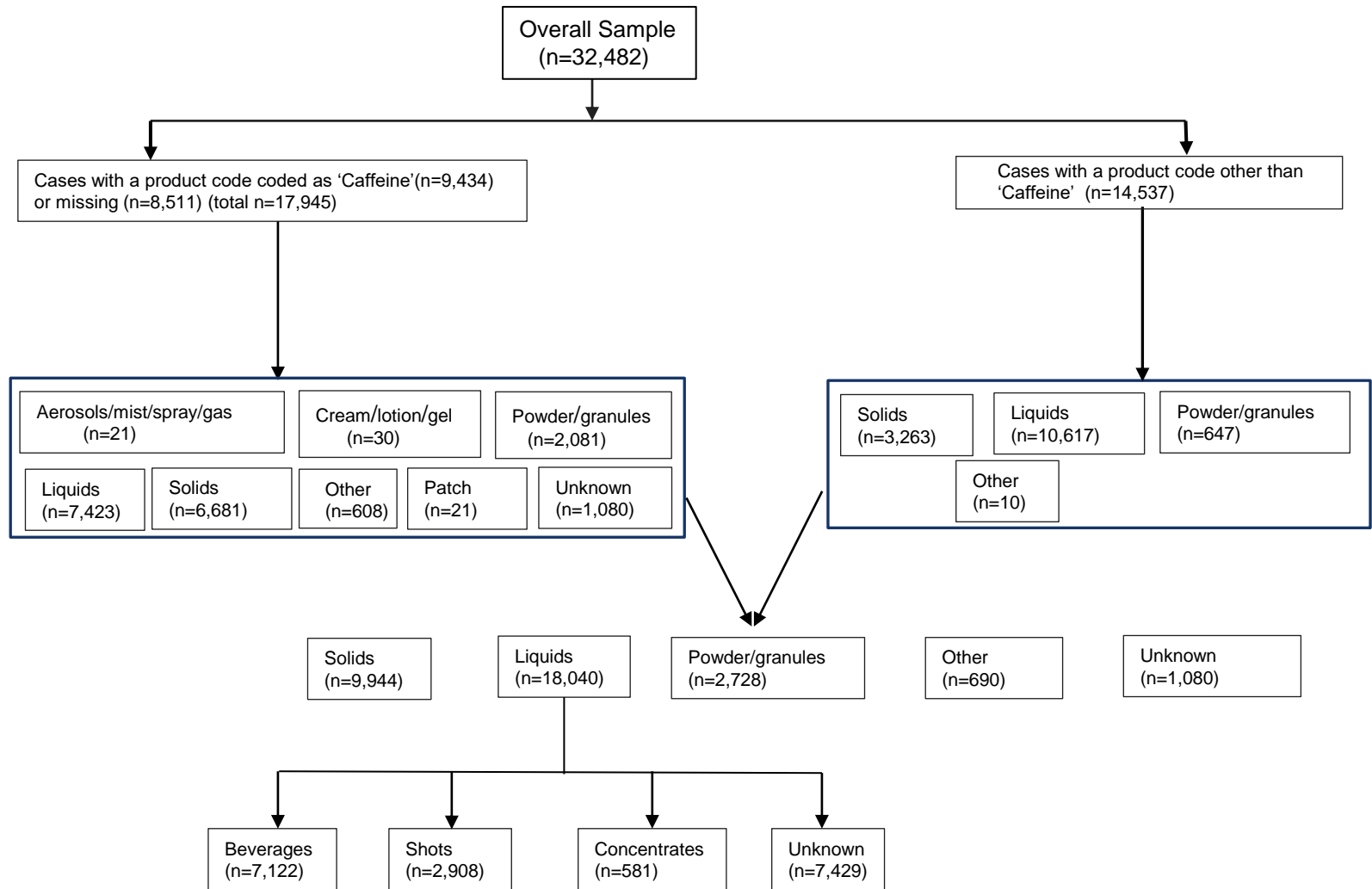

Supplement: Supplementary file 1 — Supplementary Material 1: Appendix 1. Flowchart for Assigning Formulation Categories for Caffeine Energy Products, National Poison Data System 2011-2023 [file 13181_2025_1057_MOESM1_ESM.pdf]
