# Supplementary material for: Pediatric Exposures Associated with Caffeine Energy Products Reported to United States Poison Centers, 2011–2023
Source: J Med Toxicol. 2025 Jan 31;21(2):241–52. doi: 10.1007/s13181-025-01057-w (PMC11933505; doi:10.1007/s13181-025-01057-w)
Supplement: Supplementary file 2 — Supplementary Material 2 [file 13181_2025_1057_MOESM2_ESM.docx]

**Pediatric Exposures Associated with Caffeine Energy Products Reported to United States Poison Centers, 2011-2023**

**ABSTRACT**

**Introduction:** This study investigated the characteristics and trends of pediatric exposures to caffeine energy products reported to US poison centers.

**Methods:** National Poison Data System data for caffeine energy product single-substance exposures during 2011-2023 among individuals <20 years old were analyzed.

**Results:** There were 32,482 caffeine energy product exposures reported to US poison centers with a 17.3% exposure rate increase during 2011-2023. Most exposures were among <6-year-olds (69.6%), males (56.7%), or involved liquid formulations (57.5%). Most (80.7%) were not treated in a healthcare facility; however, 1.6% were medically admitted. Teenagers 13-19 years old were more likely to be medically admitted (OR=12.74, 95% CI: 10.40-15.60) or have a serious medical outcome (OR=18.83, 95% CI: 16.88-21.01) than children <13 years old. Solid energy product formulations were more likely to be associated with a serious medical outcome (OR=1.98, 95% CI: 1.81-2.17) or medical admission (OR=5.23, 95% CI: 4.31-6.36) than other types of formulations. During the study period, exposure rates increased for liquid (34.5%) and powder/granules (632.9%) product formulations but decreased for solids (-51.5%). Among liquid formulation subcategories, the exposure rate for beverages increased (46.5%) and that for shots decreased (-86.1%).

**Conclusions:** Although most pediatric exposures to caffeine energy products reported to US poison centers were associated with no or minimal clinical effects, serious medical outcomes and medical admissions occurred. The product formulations that drove the 17% increase in the exposure rate changed during the study period. Opportunities exist to reduce the adverse effects of caffeine energy products among the pediatric population.

**Key words:** Children; Adolescents; Toxicity; Caffeine; Adverse Drug Event

**INTRODUCTION**

Caffeine, 1,3,7-trimethylxanthine, is the most consumed psychostimulant in the world [1,2]. It can improve physical and cognitive performance when consumed at safe levels [1]. Caffeine is found in coffee and cocoa beans, kola nuts, guarana, tea leaves, and other plants but can also be produced synthetically [2]. Caffeinated energy drinks were first sold in the United States (US) in 1997, followed by other caffeinated energy products, including shots, concentrates, powders, pills, and other formulations [3]. For the average nonpregnant adult, consumption of <400mg per day of caffeine is considered generally safe [1,4]. However, caffeine has been reported to cause adverse cardiac, neurologic, and gastrointestinal effects, including death [1,5-16], and these effects occur in the absence of preexisting conditions [8,17]. In a study of dietary supplement-related exposures reported to US poison centers (PCs), energy products accounted for the greatest proportion of serious medical outcomes [18]. Multiple health organizations, including the American Academy of Pediatrics, warn that there is no safe amount of energy drink consumption for children and adolescents [6,19-23] and a report from members of the US Congress warned of harmful marketing of caffeine energy drinks to adolescents [24]. Despite this, from 2008-2015, children <6 years old represented the highest percentage (42.3%) of caffeine-related exposures reported to US PCs, and youth <20 years old accounted for more than two-thirds of these exposures [25].

Researchers have previously examined caffeine-related exposures reported to PCs using the Texas Poison Center Network and the National Poison Data System (NPDS) [10,17,18,25,26]. However, the most recent study using the Texas Poison Center Network [10] examined data through only 2014, and the most recent study using national data from the NPDS examined data only through 2015 and did not subcategorize liquid formulations into beverages, shots, and concentrates [25]. In addition, to our knowledge, previous studies that analyzed PC data included individuals of all ages and did not focus exclusively on the pediatric population. Therefore, there is a need to update our knowledge with current information about this public health problem with a focus on children and adolescents.

The objective of this study was to investigate the characteristics and trends of exposures to caffeine energy products reported to US PCs from 2011 through 2023 among children and adolescents <20 years old.

**METHODS**

**Data Sources**

We analyzed data from the NPDS, which is a data warehouse maintained by America’s Poison Centers that includes substance exposures reported to all US PCs [27,28]. Specialists in Poison Information at each PC enter data in near real-time using standardized protocols for data coding and case follow-up. America’s Poison Centers also obtained detailed narratives regarding two caffeine energy product-related fatalities that occurred during the study period from the PCs that reported those fatalities; America’s Poison Centers then provided those deidentified narratives to us, which included more details about the circumstances of these events. Additionally, US census data were obtained from the US Census Bureau and used to calculate population-based exposure rates, including sex-specific and age group-specific rates [29,30]. This study was judged to be exempt by the institutional review board of the authors’ institution.

**Case Selection Criteria**

The NPDS assigns a generic code and may assign a product code to each product. The NPDS defines a generic code as “a code that represents a broad group of related products” and a product code as “a code specific to the exact substance or product by brand name, concentration, and formulation.” A generic code “allows for groupings of related product codes (each product code is assigned to only one generic code)” [31]. Generic codes for caffeine energy drinks were introduced in the NPDS in mid-2010. We therefore limited study analyses to exposures occurring in the years 2011 or after. Single-substance exposures involving caffeine energy products among individuals <20 years old reported to US PCs from January 1, 2011, to December 31, 2023, were included in the study. Exposures involving coffee, tea, and caffeinated conventional soft drinks were not included because these are not considered energy products [10]. Caffeine energy products were identified using the NPDS generic codes: 0084000 (caffeine); 0200605 (energy drinks: caffeine containing [from any source including guarana, kola nut, tea, yerba mate, cocoa, etc.]); and 0200606 (energy drinks: caffeine only [without guarana, kola nut, tea, yerba mate, cocoa, etc.]). Exposures involving energy drinks containing ethanol (n=384) were excluded from the study. Additionally, exposures were excluded if 1) the reason for exposure was “unintentional - food poisoning” (n=38) or “adverse reaction - food” (n=845), 2) the medical outcome was “confirmed non-exposure” (n=149) or “unrelated exposure” (n=535), or 3) the involved products were explicitly non-energy-related, such as diet pills, plants, or medicinal treatments (n=4,164) [31].

There were two fatalities reported during the study period that were excluded from analyses based on information contained in detailed narratives regarding these deaths provided to the NPDS by the two PCs involved. The first fatality was an 18-year-old male, who ingested an unknown quantity of powdered caffeine in his own residence with the reason for exposure coded as “unintentional - unknown.” This case was excluded because it was an “indirect report,” which means that the PC was not contacted directly about the exposure, but rather, the PC became aware of the event from another source and forwarded the information to the NPDS. The second fatality was a 12-year-old male, who ingested an unknown quantity of caffeinated powder/granules in water in his own residence with the reason for exposure coded as “adverse reaction - drug.” The case was excluded from this study because America’s Poison Centers judged the “relative contribution to fatality” of the caffeinated powder/granules as “unknown.” Deaths reported through the NPDS undergo an extensive formal review process and are given a relative contribution to fatality classification: 1) undoubtedly responsible, 2) probably responsible, 3) contributory, 4) probably not responsible, 5) clearly not responsible, or 6) unknown. America’s Poison Centers considers a fatality to be exposure-related if its fatality review team judges the substance exposure to be at least contributory (i.e., relative contribution to fatality = 1-3) [28]. The final study population included 32,482 exposures for analysis.

**Study Variables**

Variables in this study included year of exposure, sex, age group, exposure site, reason for exposure, product formulation, related clinical effects, performed therapies, highest level of health care received, and medical outcome. Age was categorized as: 1) <6 years old, 2) 6-12 years old, and 3) 13-19 years old. Exposure site was grouped as 1) residence, 2) other, and 3) unknown. Reason for exposure was categorized as 1) unintentional – general, 2) unintentional – other, 3) unintentional – unknown, 4) suspected suicide (both fatal and non-fatal), 5) intentional – misuse, 6) abuse, 7) intentional – unknown, 8) other, and 9) unknown [31].

The highest level of health care received was based on NPDS categories [31], which included 1) no treatment received at a healthcare facility, 2) treated/evaluated and released from a healthcare facility, 3) admitted to a critical care unit, 4) admitted to a non-critical care unit, 5) admitted to a psychiatric facility, 6) patient refused referral/did not arrive at a healthcare facility, and 7) unknown (which included patient lost to follow-up, left against medical advice, and unknown). Exposures with the management site coded as "unknown" were included in the unknown category for the highest level of health care received. Admissions to a critical care unit or a non-critical care unit were grouped together as “medical admissions” during analyses, thereby excluding admissions to a psychiatric facility in these analyses.

Medical outcome was based on NPDS categories [31], which included 1) no effect, 2) minor effect (minimally bothersome symptoms that generally resolve rapidly with no residual disability), 3) moderate effect (more pronounced, prolonged, or systemic than minor symptoms), 4) major effect (symptoms are life-threatening or result in significant disability or disfigurement), 5) death, 6) not followed (included not followed - minimal clinical effects possible and not followed - judged as a non-toxic exposure), and 7) unknown (included unable to follow - judged as a potentially toxic exposure). During analyses, moderate effect and major effect were grouped into the category, “serious medical outcome.”

Product formulations were categorized as 1) solids (including “tablet,” “crystals,” “capsule,” “tablet chewable,” or “lozenge/troche”), 2) liquids (including solutions), 3) powder/granules (including “powder for solution” and “powder,”), 4) other (including “aerosol/mist/spray/gas,” “cream/lotion/gel,” or “patch”), and 5) unknown (Appendix 1). These categories were assigned based on the NPDS substance product code, except for those with a product code of “caffeine.” In cases for which the product code was “caffeine” or missing, the formulation designation assigned by a Specialist in Poison Information at the PC was used to categorize the substance.

Based on the categorization used by Borron et al. [10], liquid formulations were subcategorized as 1) shots (single-dose products sold in volumes of 3 oz or less), 2) concentrates (meant for dilution in water as “water enhancers” and generally sold in volumes of 4 oz or less), 3) beverages (generally consumed in volumes of 6 oz or more), and 4) unknown. These subcategories were assigned manually by the study’s lead author based on specifications, descriptions, and photographs found on manufacturer, retailer, and energy drink-enthusiast (bevnet.com, caffeineinformer.com, bevindustry.com, overcaffeinated.org, and caffeinecontenthub.com) websites. If information about the liquid product’s subcategory type was not found online (insufficient product details, manufacturer out of business, etc.), the liquid formulation subcategory was designated as “unknown.”

**Statistical Analysis**

Analyses were conducted using IBM SPSS Statistics 29.0 (IBM Corporation, Armonk, NY) and SAS 9.4 (SAS Institute, Inc. Cary, NC). We reported descriptive statistics and population-based rates based on our study variables. Statistical analyses were performed to provide a quantitative understanding of the relationships and trends seen in this observational study. Simple linear regression was performed to determine the statistical significance of secular trends by evaluating whether the null hypothesis of slope (m)=0 could be rejected using a Student’s t-test at a significance level of α=0.05. Odds ratios (ORs) with 95% confidence intervals (CIs) were calculated to assess the magnitude of relationships between selected categories of independent variables (including age group, product formulation type, and liquid formulation subcategory type) and the dependent variables, “serious medical outcome” and “medical admission.” An OR will overestimate the magnitude of a relationship between independent and dependent variables when the outcome is not rare. Because the outcomes (i.e., serious medical outcome and medical admission) were not rare events among exposures involving suspected suicide intent, risk ratios (RRs) with 95% CIs were used to assess the magnitude of the relationships between suspected suicide and these outcomes.

**RESULTS**

**General Characteristics**

From 2011-2023, there were 32,482 single-substance exposures reported to US PCs involving caffeine energy products among children and adolescents <20 years old. More than two-thirds (69.6%) of these exposures were among children <6 years old, followed by 13-19-year-olds (20.7%) (Table 1). Males accounted for 56.7% of exposures, and 44.5% of exposures occurred in a residence.

**Reason for Exposure**

Most exposures were unintentional (82.1%), with unintentional - general exposures accounting for three-fourths (76.2%) of exposures (Table 1). However, the reason for exposure varied by age group. Most exposures among children <6 years old (98.0%) and 6-12 years old (55.9%) were associated with an unintentional - general reason. Most exposures among 13-19-year-olds were intentional (60.7%), with intentional - misuse (33.6%) being the most common reason in this age group. Of the 959 reported suspected suicides, 904 (94.3%) were among the 13-19-year-old age group; almost two-thirds (63.9%) of the 959 suspected suicides were among females and 85.7% involved solid product formulations. Suspected suicides were more likely to be associated with a serious medical outcome (RR: 8.29, 95% CI: 7.62-9.03) or medical admission (RR: 26.26, 95% CI: 22.33-30.89) than other reasons for exposure combined.

**Product Formulation**

The most common caffeine energy product formulations were liquids (57.5%) followed by solids (31.7%), which was consistent across all age groups (Table 1). The proportion of liquids was highest among children 6-12 years old (72.6%), while the proportion of solids was highest among 13-19-year-olds (42.6%). Solid formulation-related exposures were most commonly associated with a serious medical outcome (9.8%), followed by powder/granules (8.6%) and liquids (4.3%). Similarly, 3.5% of exposures to solid formulations, 1.5% of exposures to powder/granules, and 0.6% of exposures to liquids were associated with a medical admission. Solid energy product formulations were more likely to be associated with a serious medical outcome (OR: 1.98, 95% CI: 1.81-2.17) or medical admission (OR: 5.23, 95% CI: 4.31-6.36) than other types of formulations. Likewise, powder/granule exposures were more likely to be associated with a serious medical outcome (OR: 1.40, 95% CI: 1.21-1.61).

Of products identified as liquids, 67.1% were beverages (22.7% of all exposures), 27.4% were shots (9.3% of all exposures), and 5.5% were concentrates (1.9% of all exposures). Concentrate-related exposures were most commonly (5.3%) associated with a serious medical outcome among the liquid formulation subcategories, followed by shots (4.2%) and beverages (3.0%). Caffeinated energy concentrates were more likely to be associated with a serious medical outcome (OR: 1.50, 95% CI: 1.03-2.19) than other types of liquid formulations.

**Highest Level of Health Care Received and Medical Outcome**

Most caffeine energy product exposures (80.7%) did not receive treatment in a healthcare facility, while 14.7% were treated/evaluated and released and 1.6% were medically admitted (Table 1). The proportion of individuals who experienced medical admission increased with increasing age group (0.5% among children <6 years old compared with 6.3% among 13-19-year-olds). Teenagers 13-19 years old were more likely to be medically admitted (OR: 12.74, 95% CI: 10.40-15.60) than children <13 years old.

Caffeine energy product exposures were commonly associated with no effect (25.5%) or minor effect (15.6%) and 52.2% were not followed but judged to have a non-toxic exposure or minimal clinical effects possible. Another 6.8% of exposures were associated with a serious medical outcome (including 6.5% with moderate effects and 0.2% with major effects) (Table 1). The proportion of individuals who experienced a serious medical outcome increased with increasing age group (1.0% among children <6 years old compared with 27.4% among 13-19-year-olds). Teenagers 13–19 years old accounted for 78.8% of all serious medical outcomes. Compared with children <13 years old, teenagers 13–19 years old were more likely to experience a serious medical outcome (OR: 18.83, 95% CI: 16.88-21.01).

**Related Clinical Effects and Performed Therapies**

The most common related clinical effects [31] among caffeine energy product exposures were agitation (7.6%), vomiting (7.4%), tachycardia (7.1%), and nausea (7.0%), but other cardiovascular, gastrointestinal, and neurological effects occurred, including 22 reports of seizures. Therapies performed for patients in a healthcare facility included intravenous fluids (3.8%), benzodiazepines (1.5%), and 10 reports of intubation/mechanical ventilation.

**Trends**

The rate of single-substance caffeine energy product exposures among individuals <20 years old per one million US population reported to US PCs increased by 17.3% from 30.1 in 2011 to 35.3 in 2023 (m=0.309, *P*=0.015). A similar statistically significant increasing trend was observed among <6-year-olds (20.1% increase, m=1.156, *P*<0.001) and males (27.8% increase, m=0.390, *P*=0.040) over the study period (Figure 1). The rate of exposure to solid formulations decreased significantly by 51.5% from 12.8 in 2011 to 6.2 in 2023 (m= -0.464, *P*<0.001) (Figure 2). However, the rates of exposure increased non-linearly for liquids by 34.5% from 15.5 in 2011 to 20.8 in 2023 and for powder/granules by 632.9% from 0.85 in 2011 to 6.2 in 2023. Among liquid formulations, the rate of exposure involving beverages increased significantly by 46.5% from 5.7 in 2011 to 8.3 in 2023 (m=0.189, *P*<0.001), while the exposure rate for shots decreased significantly by 86.1% from 6.5 in 2011 to 0.9 in 2023 (m= -0.439, *P*<0.001) (Figure 3). There were no exposures involving concentrates reported in 2011 and 2012, and subsequently, the exposure rate remained relatively constant from 2013 to 2023 (m=0.028, *P*=0.258). The exposure rates associated with serious medical outcomes (m=0.008, *P*=0.594) and medical admissions (m=0.004, *P*=0.543) remained relatively constant throughout the study period (Figure 4).

**DISCUSSION**

The US Food and Drug Administration (FDA) has regulated caffeine as an ingredient in food since 1958 [2]. Caffeine in cola-type soft drink beverages is generally recognized as safe at levels of 200 parts per million or less, which is approximately 71 mg or less of caffeine per 12 oz; however, no similar safe level has been established for caffeine in energy products [2]. Popular energy drink products commonly contain 160 mg of caffeine in a 16 oz serving, but energy drinks with concentrations up to 300 mg per 16 oz are available. Two energy shot products identified in this study contained 200 and 230 mg of caffeine in a 1.9 oz serving [32]. In the US, manufacturers may choose to market their caffeine energy products as either foods or dietary supplements because the FDA has not established a separate regulatory category for energy products. Foods and dietary supplements have different regulatory requirements regarding ingredients, good manufacturing practices, and labeling [2,19,33]. Most caffeinated beverages are marketed as foods, which must use additives or ingredients known to be safe for their intended use, while caffeinated shots are primarily marketed as dietary supplements, which are only required to have information provided by the manufacturer supporting the safety of ingredients [24,34]. Dietary supplements are not approved by the FDA, but reporting of adverse events associated with dietary supplements is mandatory; foods are approved by the FDA but reporting of associated adverse events is not mandated [24,34].

Although caffeinated alcoholic beverages were removed from sale in 2010 after the FDA warned manufacturers against the addition of caffeine to alcoholic beverages because it was an “unsafe food additive” [35], Cappelletti et al. described US regulation of energy products, in 2015, as “one of the most lax normative frameworks” in the world because of limited oversight and restrictions [1]. In the time since, the FDA prohibited the sale of bulk powder and liquid caffeine in highly concentrated or pure forms to consumers in 2018 [36,37], but more remains to be done. The caffeinated energy product market continues to expand with gum, waffles, snacks, and candies representing new sources of caffeine consumption that, marketed as foods, may not be required to report adverse events [2]. Mandating these reports for all caffeine energy products would help provide information about changing caffeine consumption practices and risks at a population level.

There was a 17% increase in the rate of single-substance exposures among individuals <20 years old involving caffeine energy products reported to US PCs from 2011 through 2023. This demonstrated an increase from a previous study by Markon, et al. [25], who found a relatively steady rate among individuals of all ages from 2011-2015. Similar to previous studies, the rate was driven by unintentional exposures among children <6 years old and there was a male predominance [17,18,25]. Most caffeine energy product exposures in this study were associated with no or minimal effects, although 6.8% were associated with a serious medical outcome (including 6.5% with moderate effects and 0.2% with major effects) and 1.6% were medically admitted. These findings agree with the relatively low proportion of moderate and major effects reported to Texas PCs during 2010-2014 [10].

Beverages remain the most successful commercial product in the energy product market with $22 billion in sales from March of 2023 to March of 2024, a 9.9% increase over the previous year [38]. In September 2024, America’s Poison Centers announced an increase in caffeinated energy beverage-related exposures to US PCs from 2022 to 2023 [39]. Our study showed low reported exposures rates for concentrates and shots, but a consistent increase in the beverage-related exposure rate led to an overall rise in pediatric caffeine energy product exposures associated with liquid formulations during the study period. There was a higher proportion of serious medical outcomes associated with concentrates and shots than beverages, and this association between more highly concentrated caffeine energy products and serious medical outcomes agrees with findings from the smaller prior study from Texas PCs [10].

There was a 633% increase in powder/granules-related exposures during the 13-year study period. A previous study of powdered caffeine exposures reported to three PCs from 2013 to mid-2015 found that these events were uncommon [5]. In contrast, our study indicated that powdered caffeine is a growing source of exposures that was second only to solid formulations in being associated with serious medical outcomes. Unintentional overdosing may occur because of unclear dosing instructions or preparation errors by the consumer. Concentrated powder or granules may also be mistaken by a young child as a food item. While medical societies and others have highlighted their concerns regarding caffeine energy beverages, the mitigation of risks associated with legally available powder energy products is also important [20,22].

Solid caffeine energy product exposures experienced a marked decline over the 13 years of our study. Despite this, they still contributed an average of >750 exposures annually and had the highest proportion of serious medical outcomes and medical admissions of all product formulations. Solids were the formulation associated with the majority (86%) of suspected suicides. This is not a problem unique to the US. In 2004, Sweden decreased the maximum number of caffeine tablets from 250 to 30 that could be bought in a single over-the-counter purchase to help prevent suicides associated with these products; this seemed to be effective based on a subsequent observed decrease in the number of fatalities where caffeine contributed to the cause of death, although the effect was not evident until several years after the restriction was implemented [40]. Caffeine pills can currently be purchased in the US with 750 capsules (200 mg each) per bottle [41]. A similar action for solid caffeine products in the US may merit consideration.

More than two-thirds of exposures to caffeine energy products in this study were among children <6 years old, and the rate of exposure in this age group increased by 20% during the 13-year period. The high and increasing rate of exposure in this vulnerable age group indicates that caffeine energy products are accessible and attractive to young children. Consistent with recommendations of other researchers [17], caffeine energy products should not have packaging that is appealing to young children and should be kept out of the sight and reach of this age group in the home.

Teenagers 13-19 years old were 19 times more likely to experience a serious medical outcome and 13 times more likely to be medically admitted to a healthcare facility than children <13 years old in this study. These findings are consistent with those of Borron et al.[10]. Given the increased severity observed in this age group, efforts to reduce adverse events associated with caffeine energy products among teenagers are especially important. A majority of US adolescents report consuming caffeine energy drinks in their lifetime [3]. Awareness of the risks of energy drinks has been shown to be associated with a decreased likelihood of habitual use [42]. Therefore, education regarding the potential harms of caffeine energy products targeting teenagers and their parents, health care providers, teachers, coaches, and other influential contacts, may help reduce toxic exposures.

A ban on the sale of caffeine energy drinks to individuals <18 years old is an additional strategy that has been implemented in other countries, including Latvia, Lithuania, and Turkey [43,44], and has been considered in the United Kingdom [45]. In 2014, the American Beverage Association adopted voluntary guidelines against the marketing of energy drinks to children <12 years old and the marketing or sale of energy drinks in K-12 schools [46]. The American Medical Association supports a ban on the marketing of “high stimulant/caffeine drinks” to youth <18 years old [47]; however, the energy product industry does not face any mandatory marketing restrictions [48]. The absence of mandatory age restrictions on marketing of these products raises concerns, especially in the context of the substantial social media exposure among youth achieved by manufacturers [49]. This has led to recent calls for a FDA investigation by members of the US Congress [50].

**Study Limitations**

The NPDS does not capture all exposures involving caffeine energy products because it is a passive surveillance system that relies on voluntary reporting. Youth may be treated in medical settings or at home without a report being made to a PC. Therefore, this study underestimates the number of these exposures among children and adolescents. There may be reporting bias, which, for example, may lead to more serious exposures or exposures to younger children being more likely to be reported. Additionally, because exposures are self-reported, PCs and America’s Poison Centers cannot fully verify the accuracy of the data. Despite well-established data protocols and quality checks, mis-categorization or miscoding may occur. The NPDS does not use personal identifiers, so there may be repeated exposures to the same individual in the dataset. We limited our study to reports classified as single-substance exposures to improve the certainty of associations with outcomes, but laboratory testing, even if it is performed, is not reported in the NPDS, and therefore, other substances may still be involved. Limiting the study to single-substance exposures precluded the evaluation of drug-drug interactions. We excluded exposures from this study if the reason for exposure was coded as “unintentional - food poisoning” or “adverse reaction – food;” however, it is possible that some excluded exposures may have involved a caffeine-containing product. Caffeine dose and underlying medical conditions were not accounted for in study analyses. An exposure does not necessarily mean that a poisoning or overdose occurred. Our independent determination of liquid caffeine energy product subcategories had limitations, including 1) some product information was not found online, and 2) we relied on webpages and images to make determinations that may not have been accurate; therefore, mis-categorization of product subcategory was possible. A consistent coding framework in the NPDS to differentiate between these product subtypes would provide a better long-term solution for improved monitoring of caffeine energy product subtype exposures and associated outcomes. Despite its limitations, the NPDS provides an extensive, standardized database that has been widely used to investigate toxic exposures at the national level.

**CONCLUSIONS**

Although most caffeine energy product exposures among children and adolescents reported to US PCs were associated with no or minimal clinical effects, serious medical outcomes and medical admissions occurred. The caffeine energy product exposure rate increased 17% from 2011 through 2023, and the product formulations driving the observed trends changed over time, including increases in powdered caffeine products and beverages. Opportunities exist to reduce the adverse effects of caffeine energy products among the pediatric population.

**Acknowledgments**

None

**REFERENCES**

1. Cappelletti S, Piacentino D, Sani G, et al. Caffeine: Cognitive and physical performance enhancer or psychoactive drug? *Curr Neuropharmacol*. 2015;13(1):71-88. doi:10.2174/1570159X13666141210215655

2. Rosenfeld LS, Mihalov JJ, Carlson SJ, et al. Regulatory status of caffeine in the United States. *Nutr Rev*. 2014;72 23-33. doi:10.1111/nure.12136

3. Miller KE, Dermen KH, Lucke JF. Caffeinated energy drink use by US adolescents aged 13-17: A national profile. *Psychol Addict Behav*. 2018;32(6):647-659. doi:10.1037/adb0000389

4. European Food Safety Authority. Caffeine. Updated Sept 29, 2017. Accessed Jun 10, 2024. Available from: <https://www.efsa.europa.eu/sites/default/files/corporate_publications/files/efsaexplainscaffeine150527.pdf>

5. Beauchamp GA, Johnson AR, Crouch BI, et al. A retrospective study of clinical effects of powdered caffeine exposures reported to three US Poison Control Centers. *J Med Toxicol*. 2016;12(3):295-300. doi:10.1007/s13181-016-0545-9

6. Seifert SM, Schaechter JL, Hershorin ER, et al. Health effects of energy drinks on children, adolescents, and young adults. *Pediatrics*. 2011;127(3):511-528. doi:10.1542/peds.2009-3592

7. Gunja N, Brown JA. Energy drinks: Health risks and toxicity. *Med J Aust*. 2012;196(1):46-49. doi:10.5694/mja11.10838

8. Li P, Haas NA, Dalla-Pozza R, et al. Energy drinks and adverse health events in children and adolescents: A literature review. *Nutrients*. 2023;15(11):2537. doi:10.3390/nu15112537

9. Moussa M, Hansz K, Rasmussen M, et al. Cardiovascular effects of energy drinks in the pediatric population. *Pediatr Emerg Care*. 2021;37(11):578-582. doi:10.1097/PEC.0000000000002165

10. Borron SW, Watts SH, Herrera J, et al. Energy drink exposures reported to Texas poison centers: Analysis of adverse incidents in relation to total sales, 2010-2014. *Regul Toxicol Pharmacol*. 2018;97:1-14. doi:10.1016/j.yrtph.2018.05.008

11. Oberhoffer FS, Li P, Jakob A, et al. Energy drinks: Effects on blood pressure and heart rate in children and teenagers. A randomized trial. *Front Cardiovasc Med*. 2022;9:862041. doi:10.3389/fcvm.2022.862041

12. Mansour B, Amarah W, Nasralla E, et al. Energy drinks in children and adolescents: Demographic data and immediate effects. *Eur J Pediatr*. 2019;178(5):649-656. doi:10.1007/s00431-019-03342-7

13. Fletcher EA, Lacey CS, Aaron M, et al. Randomized controlled trial of high-volume energy drink versus caffeine consumption on ECG and hemodynamic parameters. *J Am Heart Assoc*. 2017;6(5):e004448. doi:10.1161/JAHA.116.004448

14. Cappelletti S, Piacentino D, Fineschi V, et al. Caffeine-related deaths: Manner of deaths and categories at risk. *Nutrients*. 2018;10(5):611. doi:10.3390/nu10050611

15. Ajibo C, Van Griethuysen A, Visram S, et al. Consumption of energy drinks by children and young people: A systematic review examining evidence of physical effects and consumer attitudes. *Public Health*. 2024;227:274-281. doi:10.1016/j.puhe.2023.08.024

16. Temple JL, Bernard C, Lipshultz SE, et al. The safety of ingested caffeine: A comprehensive review. *Front Psychiatry*. 2017;8:80. doi:10.3389/fpsyt.2017.00080

17. Seifert SM, Seifert SA, Schaechter JL, et al. An analysis of energy-drink toxicity in the National Poison Data System. *Clin Toxicol*. 2013;51(7):566-574. doi:10.3109/15563650.2013.820310

18. Rao N, Spiller HA, Hodges NL, et al. An increase in dietary supplement exposures reported to US poison control centers. *J Med Toxicol*. 2017;13(3):227-237. doi:10.1007/s13181-017-0623-7

19. Bashir D, Reed-Schrader E, Olympia RP, et al. Clinical symptoms and adverse effects associated with energy drink consumption in adolescents. *Pediatr Emerg Care*. 2016;32(11):751-755. doi:10.1097/PEC.0000000000000703

20. Committee on Nutrition and the Council on Sports Medicine and Fitness. Sports drinks and energy drinks for children and adolescents: Are they appropriate? *Pediatrics*. 2011;127(6):1182-1189. doi:10.1542/peds.2011-0965

21. Hampton T. Energy drinks pose worrisome risks to adolescents’ cardiovascular health. *Circulation*. 2016;134(14):1052-1053. doi:10.1161/CIRCULATIONAHA.116.025039

22. Jagim AR, Harty PS, Tinsley GM, et al. International society of sports nutrition position stand: Energy drinks and energy shots. *J Int Soc Sports Nutr*. 2023;20(1):2171314. doi:10.1080/15502783.2023.2171314

23. Schering S. Children should avoid drinks with sugar, caffeine. AAP News. Updated Dec 1, 2023. Accessed Jun 10, 2024. Available from: <https://publications.aap.org/aapnews/news/27276/Children-should-avoid-drinks-with-sugar-caffeine>

24. Staff of Senator Edward J. Markey, Richard J. Durbin, and Richard Blumenthal. Buzz Kill. Updated Dec 30, 2014. Accessed Jun 10, 2024; 30]. Available from: <https://www.markey.senate.gov/imo/media/doc/2014-12-30-Report_BuzzKill_EnergyDrinks_ScreenV.pdf>

25. Markon AO, Jones OE, Punzalan CM, et al. Caffeinated energy drinks: Adverse event reports to the US Food and Drug Administration and the National Poison Data System, 2008 to 2015. *Public Health Nutr*. 2019;22(14):2531-2542. doi:10.1017/S1368980019001605

26. Seifert SA, Arheart KL, Franco VI, et al. Energy drink exposures in the National Poison Data System: Epidemiology and clinical effects [Abstract]. *Circulation*. 2018;130:A14841. Accessed Jun 10, 2024. <https://www.ahajournals.org/doi/10.1161/circ.130.suppl_2.14841>.

27. America’s Poison Centers. National Poison Data System. Updated Dec 18, 2023. Accessed Jun 10, 2024. Available from: <https://poisoncenters.org/national-poison-data-system>

28. Gummin DD, Mowry JB, Beuhler MC, et al. 2022 Annual report of the National Poison Data System (NPDS) from America’s Poison Centers: 40th Annual report. *Clin Toxicol*. 2023;61(10):717-939. doi:10.1080/15563650.2023.2268981

29. US Census Bureau. Annual estimates of the resident population by single year of age and sex for the United States: April 1, 2010 to July 1, 2020. Updated Oct 8, 2021. Accessed Aug 29, 2024. Available from: <https://www.census.gov/programs-surveys/popest/technical-documentation/research/evaluation-estimates/2020-evaluation-estimates/2010s-national-detail.html>

30. US Census Bureau. Annual estimates of the resident population by single year of age and sex for the United States: April 1, 2020 to July 1, 2023. Updated Jun 25, 2024. Accessed Aug 29, 2024. Available from: <https://www.census.gov/data/tables/time-series/demo/popest/2020s-national-detail.html>

31. America’s Poison Centers. NPDS coding users’ manual. Updated Mar 21, 2023. Accessed Sept 13, 2024. Available from: <https://www.npds.us/Help/NPDS%20Coding%20User%20Manual%20(June%202023).pdf>

32. Anonymous. Caffeine chart. Center for Science in the Public Interest. Accessed Dec 6, 2024. Available from: <https://www.cspinet.org/caffeine-chart#soda>

33. Mattia A. Regulatory status of caffeine. Updated Aug 16, 2013. Accessed Jun 10, 2024. Available from: <https://ods.od.nih.gov/pubs/energydrinks2013/Mattia.pdf>

34. US Food and Drug Administration. New dietary ingredients in dietary supplements - Background for industry. Updated Feb 9, 2023. Accessed Jul 25, 2024. Available from: <https://www.fda.gov/food/new-dietary-ingredient-ndi-notification-process/new-dietary-ingredients-dietary-supplements-background-industry>

35. US Food and Drug Administration. Caffeinated alcoholic beverages. Updated Nov 17, 2010. Accessed Jun 13, 2024. Available from: <https://www.fda.gov/food/food-additives-petitions/caffeinated-alcoholic-beverages>

36. US Food and Drug Administration. FDA takes step to protect consumers against dietary supplements containing dangerously high levels of extremely concentrated or pure caffeine. Updated Apr 12, 2018. Accessed Jun 10, 2024. Available from: <https://www.fda.gov/news-events/press-announcements/fda-takes-step-protect-consumers-against-dietary-supplements-containing-dangerously-high-levels>

37. US Food and Drug Administration. FDA warns consumers about pure and highly concentrated caffeine. Updated Apr 13, 2018. Accessed Jun 10, 2024. Available from: <https://www.fda.gov/food/information-select-dietary-supplement-ingredients-and-other-substances/fda-warns-consumers-about-pure-and-highly-concentrated-caffeine>

38. Nielsen IQ. Navigating the dynamic energy drink market: 4 key trends. Nielsen Consumer LLC. Updated May 8, 2024. Accessed Jul 2, 2024. Available from: <https://nielseniq.com/global/en/insights/education/2024/energy-drink-trends/>

39. ABC Good Morning America. Poison control centers report spike in calls about kids and energy drinks. Updated Sept 17, 2024. Accessed Sept 17, 2024. Available from: <https://www.goodmorningamerica.com/family/story/poison-control-centers-report-spike-calls-kids-energy-113657994>

40. Lundqvist KM, Ahlner J. Caffeine fatalities--do sales restrictions prevent intentional intoxications? *Clin Toxicol*. 2010;48(4):354-358. doi:10.3109/15563650903586752

41. Johnson J, Davenport S. What are some of the best caffeine pills? MedicalNewsToday. Updated Oct 13, 2022. Accessed Dec 6, 2024. Available from: <https://www.medicalnewstoday.com/articles/best-caffeine-pills#_noHeaderPrefixedContent>

42. Gallimberti L, Buja A, Chindamo S, et al. Energy drink consumption in children and early adolescents. *Eur J Pediatr*. 2013;172(10):1335-1340. doi:10.1007/s00431-013-2036-1

43. Iversen KL, Arnesen E, Meltzer HM, et al. Children and adolescents need protection against energy drinks. *Tidsskr Nor Laegeforen*. 2018;138(14 ). doi: 10.4045/tidsskr.18.0585

44. Hürriyet Daily News. Turkey bans energy drink sales for under-18s. Updated Jun 30, 2017. Accessed Jun 11, 2024. Available from: <https://www.hurriyetdailynews.com/turkey-bans-energy-drink-sales-for-under-18s-114975>

45. The Labour Party. Labour’s Child Health Action Plan will create the healthiest generation of children ever. Updated Jun 10, 2024. Accessed Jun 11, 2024. Available from: <https://labour.org.uk/updates/stories/labours-child-health-action-plan-will-create-the-healthiest-generation-of-children-ever/>

46. American Beverage Association. ABA guidance for the responsible labeling and marketing of energy drinks. Updated Apr 30, 2014. Accessed Jul 26, 2024. Available from: <https://aba-bigtree.s3.amazonaws.com/files/resources/abaguidancefortheresponsiblelabelingandmarketingof.pdf>

47. American Medical Association. Hazards of energy beverages - Their abuse and regulation D-150.976. Reaffirmed 2023. Updated 2023. Accessed Aug 29, 2024. Available from: <https://policysearch.ama-assn.org/policyfinder/detail/energy%20drinks?uri=%2FAMADoc%2Fdirectives.xml-0-258.xml>

48. Kraak VI, Davy BM, Rockwell MS, et al. Policy recommendations to address energy drink marketing and consumption by vulnerable populations in the United States. *J Acad Nutr Diet*. 2020;120(5):767-777. doi:10.1016/j.jand.2020.01.013

49. Kent MP, Pauzé E, Roy E, et al. Children and adolescents' exposure to food and beverage marketing in social media apps. *Pediatr Obes*. 2019;14(6):e12508. doi:10.1111/ijpo.12508

50. Schumer CE. Letter to FDA Commissioner, Dr. Robert Califf. Majority Leader Schumer demands FDA investigate ‘Prime’ for absurd caffeine content and marketing targeting kids on social media; Schumer warns parents that summer’s hottest drink has so much caffeine that it puts Red Bull to shame. Updated Press Release July 10, 2023. Accessed Aug 29, 2024. Available from: <https://www.democrats.senate.gov/newsroom/press-releases/majority-leader-schumer-demands-fda-investigate-prime-for-absurd-caffeine-content-and-marketing-targeting-kids-on-social-media-schumer-warns-parents-that-summers-hottest-drink-has-so-much-caffeine-that-it-puts-red-bull-to-shame>

**FIGURE TITLES / LEGENDS**

**Figure 1.** Annual Rate of Pediatric Exposures Associated with Caffeine Energy Products Reported to United States Poison Centers by Age Group, National Poison Data System 2011-2023

**Figure 2**. Annual Rate of Pediatric Exposures Associated with Caffeine Energy Products Reported to United States Poison Centers by Product Formulation, National Poison Data System 2011-2023

**Figure 3.** Annual Rate of Pediatric Exposures Associated with Caffeine Energy Products Reported to United States Poison Centers by Liquid Formulation Subgroups, National Poison Data System 2011-2023

**Figure 4**. Annual Rate of Pediatric Exposures Involving Caffeine Energy Products Reported to United States Poison Centers Associated with a Serious Medical Outcome or Medical Admission, National Poison Data System 2011-2023

**TABLE TITLE / LEGEND**

**Table 1.** Characteristics of Pediatric Exposures Associated with Caffeine Energy Products Reported to United States Poison Centers by Age Group, National Poison Data System 2011-2023

**APPENDIX TITLE / LEGEND**

**Appendix 1.** Flowchart for Assigning Formulation Categories for Caffeine Energy Products, National Poison Data System 2011-2023
